# The medulla controls effector primed γδT‐cell development in the adult mouse thymus

**DOI:** 10.1002/eji.202350388

**Published:** 2023-03-28

**Authors:** Kieran D. James, Andrea J. White, William E. Jenkinson, Graham Anderson

**Affiliations:** ^1^ Institute of Immunology and Immunotherapy University of Birmingham Birmingham UK

**Keywords:** γδT cells, CCL21, Medulla, Thymus

## Abstract

γδT cells are produced in the thymus throughout life and provide immunity at epithelial‐rich sites. Unlike conventional αβT cells, γδT‐cell development involves intrathymic acquisition of effector function, with priming for either IL17 or IFN‐γ production occurring during embryonic or adult life, respectively. How the thymus controls effector‐primed γδT‐cell generation in adulthood is poorly understood. Here, we distinguished de novo γδT cells from those undergoing thymus recirculation and/or retention using Rag2GFP mice alongside markers of maturation/effector priming including CD24, CD25, CD73, and IFN‐γ, the latter by crossing with IFN‐γ^YFP^ GREAT mice. We categorize newly developing γδT‐cells into an ordered sequence where CD25^+^CD73^−^IFN‐γ^YFP−^ precursors are followed sequentially by CD25^−^CD73^+^IFN‐γ^YFP−^ intermediates and CD25^−^CD73^+^IFN‐γ^YFP+^ effectors. To determine intrathymic requirements controlling this sequence, we examined γδT‐cell development in *Relb^−/−^* thymus grafts that lack medullary microenvironments. Interestingly, medulla deficiency did not alter CD25^+^ γδT‐cell precursor generation, but significantly impaired development of effector primed stages. This impact on γδT‐cell priming was mirrored in *plt/plt* mice lacking the medullary chemoattractants CCL19 and CCL21, and also *Ccl21a^−/−^* but not *Ccl19^−/−^* mice. Collectively, we identify the medulla as an important site for effector priming during adult γδT‐cell development and demonstrate a specific role for the medullary epithelial product CCL21 in this process.

## Graphical abstract

How γδT cells undergo effector priming during their intrathymic development is unclear. In this study, we show that in adult mice, the thymus medulla is essential for the development of IFN‐γ^+^ effector γδT cells and demonstrate a specific role for CCL21, but not CCL19, in this process.

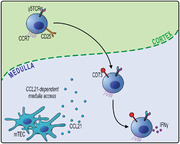

## Introduction

In order to establish and maintain an effective immune system, the thymus supports the development of both αβT‐cell receptor (TCR)^+^ and γδTCR^+^ T cells throughout multiple stages of life. For conventional αβT‐cell development, interactions with cortical thymic epithelial cells and medullary thymic epithelial cells (mTEC) result in the generation of mature CD4^+^ and CD8^+^ αβTCR^+^ thymocytes that are exported from the thymus and populate the periphery as recent thymus emigrants. However, while intrathymic events enable αβT‐cells to respond to TCR stimulation via proliferation and cytokine production, they are exported as functionally naïve cells and require additional stimulatory signals in peripheral tissues to acquire effector functions [1]. This contrasts to the process of intrathymic γδT‐cell development. Indeed, in both the fetal and adult thymus, some γδTCR^+^ thymocytes acquire effector functions as part of their intrathymic development prior to their export, often termed “intrathymic effector preprogramming”, which is a key feature of their innate‐like functions [2]. Interestingly, the fetal thymus supports the development of both IL17‐producing and IFN‐γ‐producing γδT‐cells. While the adult thymus is unable to generate IL17‐producing γδT cells, it continues to support the development of IFN‐γ‐producing effector primed γδT cells [3, 4, 5].

Importantly, the intrathymic requirements for effector priming during γδT‐cell development in the adult thymus are poorly understood. This is perhaps at least in part due to the complexity of the adult intrathymic γδT‐cell pool. For example, recent studies show this population is developmentally heterogeneous, consisting of a mixture of immature γδ thymocytes that represent de novo γδT‐cell development, and fully mature γδT‐cells that have completed their maturation and not undergone thymic export, and so represent long‐term thymus‐resident cells [6]. As such, understanding the regulation of γδ thymocyte development in the adult thymus, including intrathymic effector priming, is hindered by a lack of approaches to accurately define and study stages in de novo effector γδT‐cell development.

To address this, we used combinations of Rag2GFP and IFN‐γ^YFP^ GREAT reporter mice to study intrathymic γδ thymocyte development in the adult thymus and determine the role of epithelial microenvironments in this process. First, we show that de novo γδT‐cell development can be identified by expression of CD24 and Rag2GFP, and it involves production of a subset defined by expression of CD73, a marker of γδTCR engagement and commitment to the γδT‐cell lineage [7]. Using IFN‐γ^YFP^ GREAT mice, we identify a subset of IFN‐γ^YFP+^ cells within newly produced CD73^+^ γδthymocytes. Furthermore, by analyzing the requirement for thymic microenvironments using medullary deficient *Relb^−/−^* thymus transplants, we observe a block in the intrathymic development of CD73^+^ and CD73^+^IFN‐γ^YFP+^ γδ thymocytes. Interestingly, this requirement for the thymus medulla mapped to a requirement for CCR7 chemokine receptor ligands, and specifically the medullary chemoattractant CCL21 but not CCL19. Taken together, our study identifies an important role for the adult thymic medulla during γδ thymocyte effector priming and highlights a selective requirement for the chemokine CCL21 in this process.

## Results and discussion

### Identifying stages in effector primed γδ T‐cell development in adult mouse thymus

**Figure 1:**
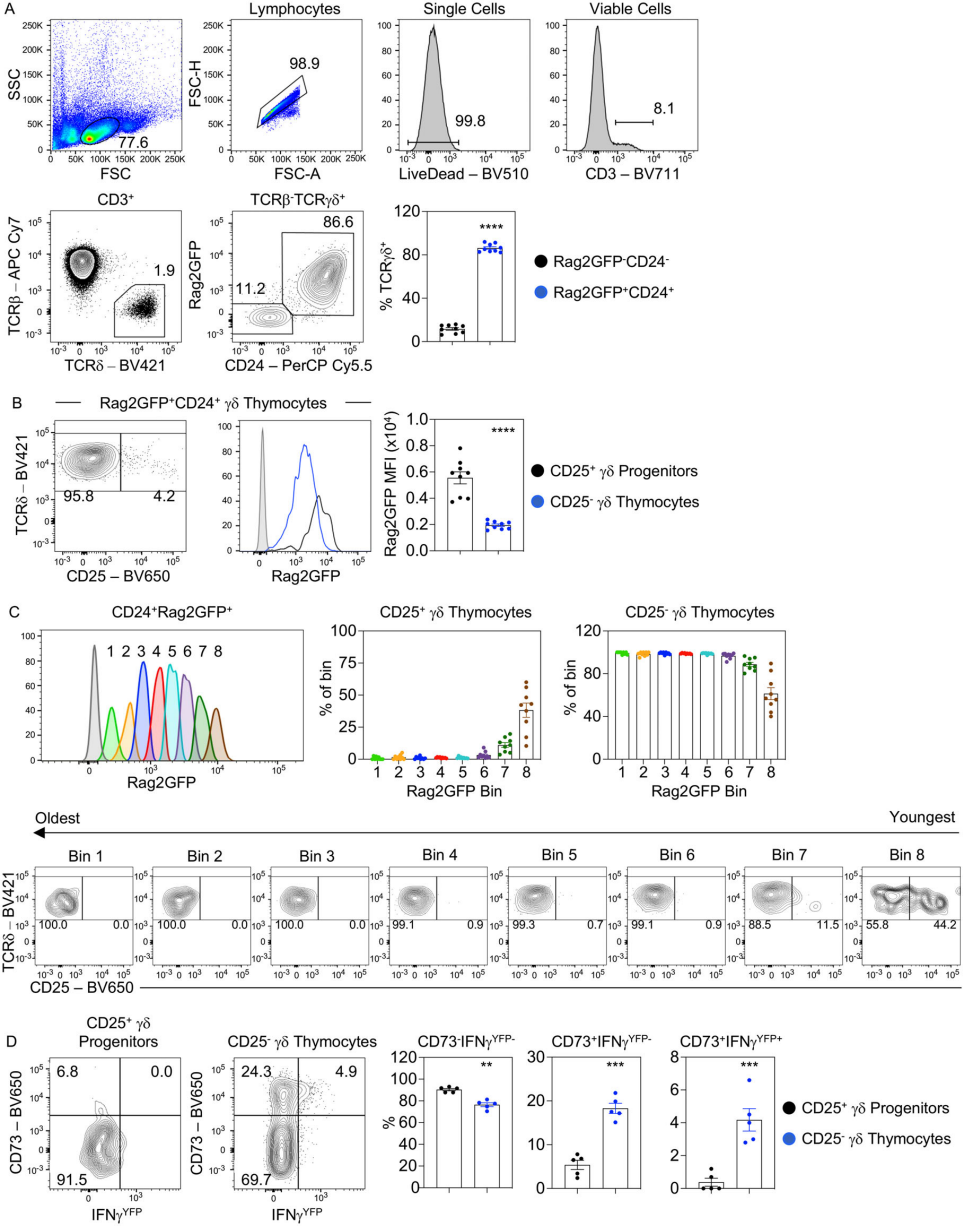
Identifying newly produced γδ thymocyte populations in the adult mouse thymus. (A) Gating strategy to identify CD3^+^TCRβ^−^TCRγδ^+^ γδ thymocytes in WT C57BL/6 mice crossed to Rag2GFP and frequencies of Rag2GFP^+^CD24^+^ γδ thymocytes were calculated (*n* = 9). (B) CD25 expression by Rag2GFP^+^CD24^+^ γδ thymocytes. The histogram shows the Rag2GFP expression levels by Rag2GFP^+^CD24^+^CD25^+^ γδ progenitors (black) and Rag2GFP^+^CD24^+^CD25^−^ (blue) γδ thymocytes and the calculated mean fluorescence intensity (MFI). Gray histograms indicate nonfluorescent control cells. (C) Bins of Rag2GFP expression within Rag2GFP^+^CD24^+^ γδ thymocytes are displayed from lowest Rag2GFP expression (Bin 1) to highest Rag2GFP expression (Bin 8) and the cells within each bin are plotted for TCRδ and CD25. The frequency of CD25^+^ and CD25^−^ within these bins is displayed in the graphs on the upper right. (D) WT C57BL/6 mice were crossed to IFN‐γ^YFP^ GREAT adult reporter mice (*n* = 5) and the representative plots show CD73 and IFN‐γ^YFP^ expression in CD24^+^CD25^+^ and CD24^+^CD25^−^ γδ thymocytes. The percentages of CD73^+^IFN‐γ^−^, CD73^+^IFN‐γ^+^, and CD73^−^IFN‐γ^−^ within the CD24^+^CD25^−^ γδ are shown in bar chart in (C). In all cases, error bars represent mean ± SEM. For statistical analysis, an unpaired Student's *t*‐test was used, where *p < 0.05; **p < 0.01; ***p < 0.001; ****p < 0.0001. Flow cytometry data representative of at least three independent experiments.

The ability to study regulation of Τ‐cell development in adult mice requires direct identification of immature thymocytes undergoing de novo intrathymic development. This is of particular significance for study of effector γδT‐cell development, as recent findings show the adult thymus contains subsets of mature, IL17, and IFN‐γ‐producing γδT cells that represent long‐term thymus residents [6]. Consistent with this, analysis of γδT‐cell development in adult Rag2GFP reporter mice, where the presence and amount of GFP identifies the relative age of different thymocyte populations [8], identified a dominant GFP^+^ γδ thymocyte population that represents cells undergoing development, and an additional smaller population of GFP^−^ cells that represents mature γδT cells (Fig. 1A). Importantly, patterns of GFP expression in γδ thymocytes fully overlap with expression of CD24, indicating that analysis of either CD24 or GFP is an appropriate and useful means to distinguish de novo γδ thymocyte development from long‐term resident cells (Fig. 1A).

To study early stages in γδ thymocyte development, we analyzed GFP^+^CD24^+^ cells for expression of CD25, a marker previously demonstrated to identify early γδTCR^+^ progenitors [9] (Fig. 1B). Within Rag2GFP^+^CD24^+^ γδ thymocytes, CD25^+^ cells had the highest GFP levels compared to CD25^−^ cells (Fig. 1B). Additionally, when using an approach previously used to study the developmental progression of regulatory αβT cells [10], when the total Rag2GFP^+^CD24^+^ γδ thymocyte population was separated into different ages within “bins” of Rag2GFP expression, we found the CD25^+^ population was dominant in the earliest bins (highest Rag2GFP expression) and absent in the older bins (lowest Rag2GFP expression), consistent with their early progenitor phenotype (Fig. 1C). Moreover, when we examined expression of CD73, an indicator of γδTCR signaling and γδ linegae commitment, together with IFN‐γ expression in IFN‐γ reporter GREAT mice [11], we saw that CD25^+^ γδ thymocytes uniformly lacked IFN‐γ^YFP^ expression, while a small proportion of cells expressed low levels of CD73 (Fig. 1D). In contrast, CD24^+^CD25^−^ cells contained cells with high levels of CD73 expression, with CD73^+^ cells containing both IFN‐γ^YFP−^ and IFN‐γ^YFP+^ subsets (Fig. 1C). Consistent with this, the older bins of the CD25^−^ population were enriched for CD73^+^ γδ thymocytes (Supporting information Fig. S1). Collectively, these findings are consistent with the immature progenitor status of CD25^+^ cells, with the presence of a small but detectable population of CD73^+^ cells perhaps suggesting that induction of effector priming is initiated within this early CD25^+^ stage. Furthermore, they also define stages in the intrathymic effector priming of adult γδ thymocytes, with the presence of CD73^+^IFN‐γ^YFP−^ and CD73^+^IFN‐γ^YFP+^ subsets within the more mature CD25^−^ γδ fraction, suggesting a developmental sequence in which CD25^+^ progenitors give rise to CD73^+^ cells, which then acquire IFN‐γ expression.

### The thymus medulla controls intrathymic effector γδ T‐cell development

**Figure 2:**
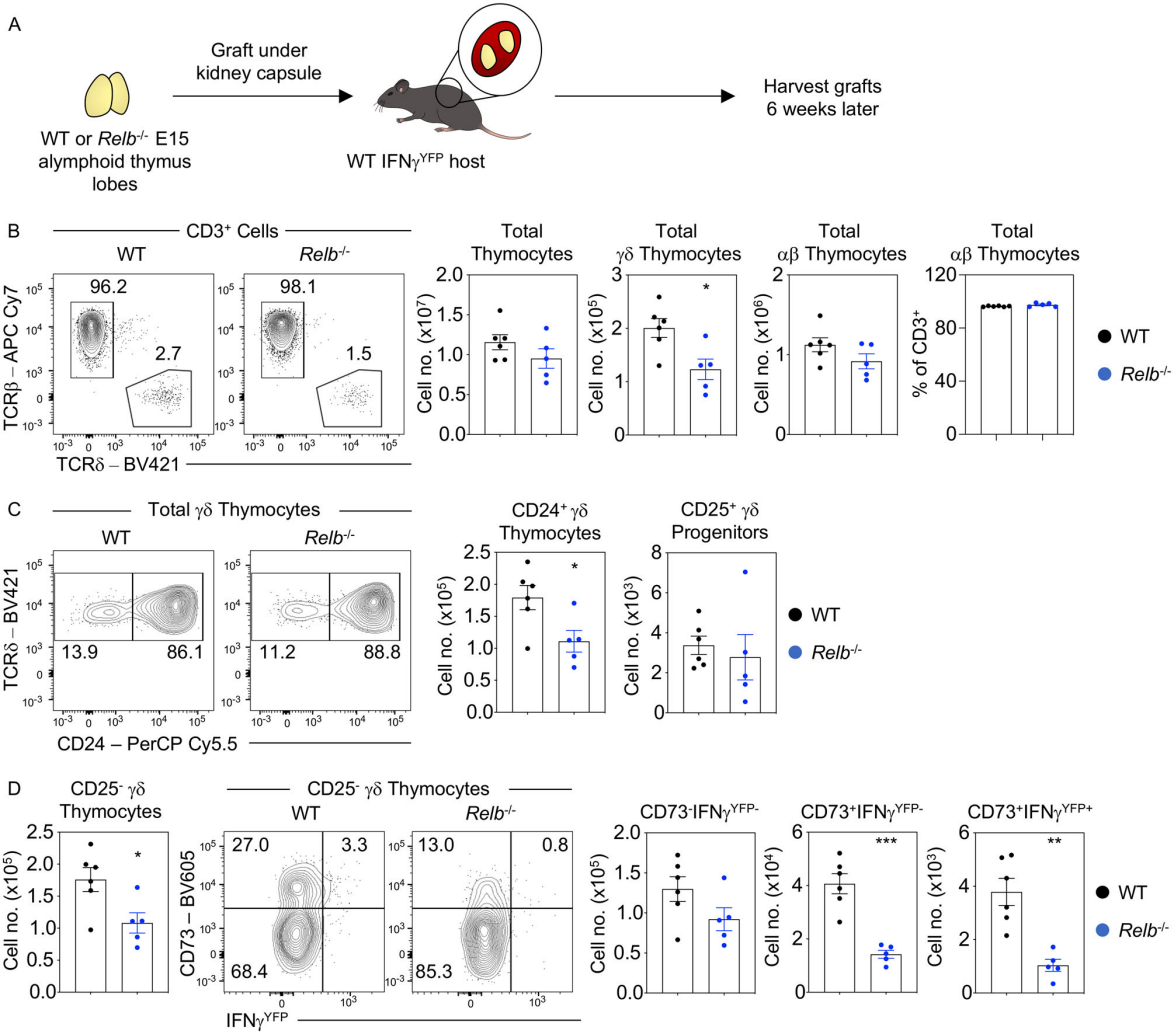
γδ effector priming is dependent on the thymus medulla. (A) Analysis of TCRγδ^+^ thymocyte development in C57BL/6 WT (WT, black, *n* = 6) and *Relb‐*deficient (*Relb^−/−^*, blue, *n* = 5) embryonic embryonic day 15 alymphoid thymus lobes that have been grafted under the capsule of the kidney of adult C57BL/6 WT IFN‐γ^YFP^ hosts and harvested 6 weeks following surgery. (B) Representative flow cytometry plots and frequencies of total thymic cellularity, TCRβ^+^ and TCRγδ^+^ thymocytes. (C) TCRγδ^+^CD24^+^ newly produced γδ thymocytes and break down into subsequent populations of CD25^+^ γδ progenitors and CD25^−^ cells. (D) Representative plots of CD73 and IFN‐γ^YFP^ expression within the TCRγδ^+^CD24^+^CD25^−^ population to identify CD73^−^IFN‐γ^YFP−^ uncommitted/naïve, CD73^+^IFN‐γ^YFP−^ effector committed, and CD73^+^IFN‐γ^YFP+^ effector γδ thymocytes. In all cases, error bars represent mean ± SEM. For statistical analysis, an unpaired Student's *t*‐test was used, where *p < 0.05; **p < 0.01; ***p < 0.001; ****p < 0.0001. All data shown are representative of two independent experiments.

The thymus medulla is essential in supporting the distinct stages of development of αβT‐cell development. In particular, the medulla controls the production of αβT‐cell subsets that acquire effector functions intrathymically, including cytokine producing invariant NKT‐cells, and immunoregulatory Foxp3^+^ regulatory T cells (Treg) [12]. This requirement is perhaps most evident from studies demonstrating defective NKT‐cell and Treg development in the thymus of *Relb^−/−^* mice, where medullary thymic microenvironments are absent [13]. To investigate the possible role of the medulla during the generation of effector primed γδT‐cells in the adult thymus, we performed thymus transplant experiments in which alymphoid thymus lobes from either WT or medulla‐deficient *Relb^−/−^* mice were grafted under the kidney capsule of adult IFN‐γ^YFP^ GREAT reporter host mice (Fig. 2A). In *Relb* ^−/−^ grafts, while total αβTCR^+^ thymocytes showed no significant reduction, total γδTCR^+^ thymocytes were reduced (Fig. 2B), and this loss was reflected in a significant reduction in CD24^+^ γδTCR^+^ thymocytes (Fig. 2C). Further analysis of newly produced CD24^+^ γδ thymocytes showed that while CD25^+^ precursors were not significantly altered (Fig. 2C), there was a reduction in the CD25^−^ fraction of CD24^+^ γδ thymocytes in *Relb^−/−^* grafts (Fig. 2D). This was accompanied by a substantial loss of CD73^+^ effector primed γδ thymocytes cells, with a reduction in CD73^+^IFN‐γ^YFP−^ cells and an almost complete loss of the CD73^+^IFN‐γ^YFP+^ population (Fig. 2D). Together, these findings suggest that effector priming during adult γδT‐cell development is dependent on signals from the thymus medulla.

### Access to the medulla via CCR7‐CCL21 regulates effector γδ T‐cell development

**Figure 3:**
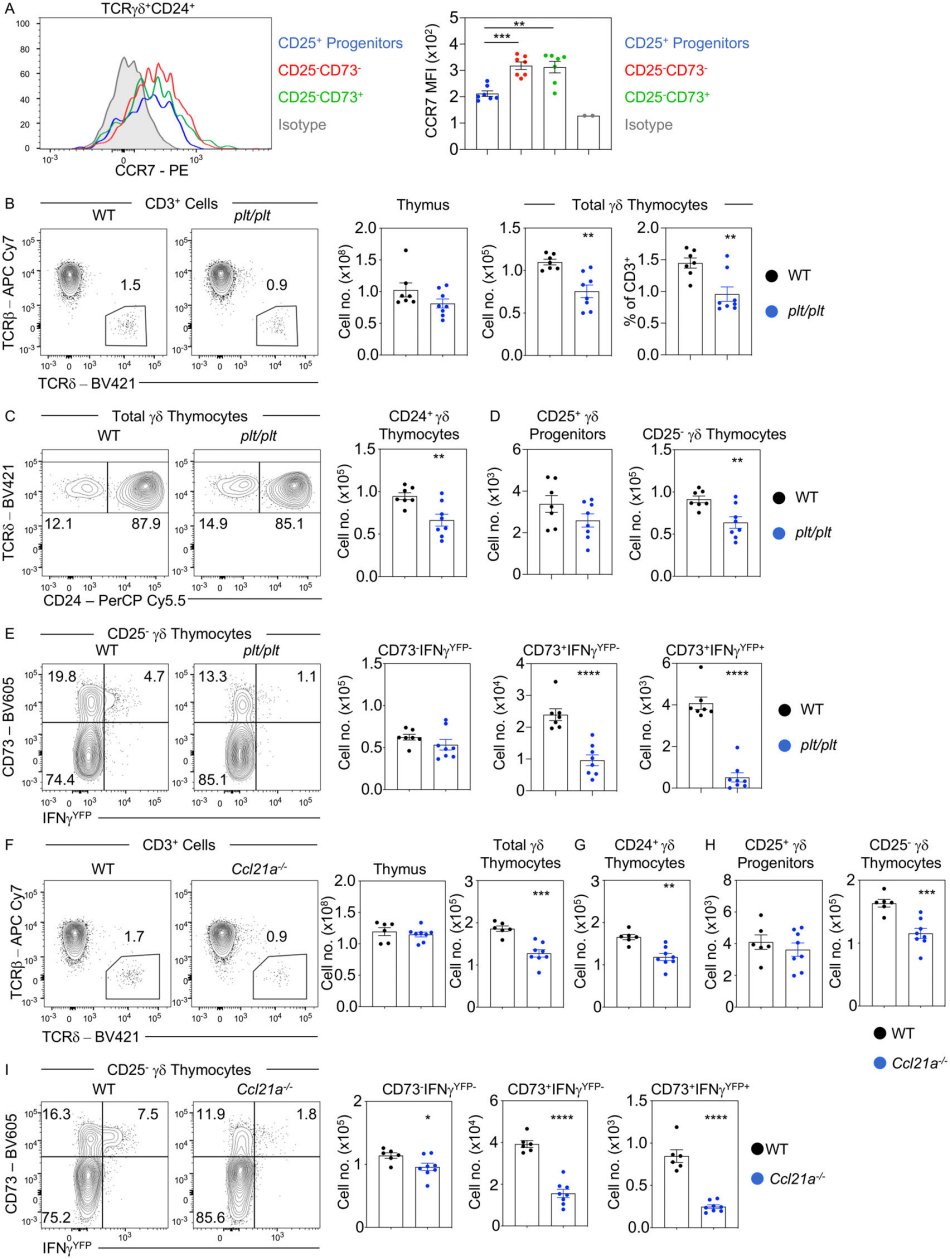
Access to the thymus medulla via CCL21a is essential for γδ effector priming during their intrathymic development. (A) Histogram of CCR7 expression by different subsets of CD24^+^ γδ thymocytes and the calculated mean fluorescence intensity (MFI) of CCR7 is shown in the bar chart on the right (*n* = 7). Gray histograms indicate isotype control staining. (B) Representative flow cytometry of identifying γδ thymocytes and frequencies of total thymus and TCRγδ^+^ cellularities in C57BL/6 WT IFN‐γ^YFP^ (WT, black, *n* = 7) and *plt/plt* IFN‐γ^YFP^ (*plt/*plt, blue, *n* = 8) adult mouse thymus. (C) Representative flow cytometry of CD24 expression on TCRγδ^+^ thymocytes and the total number of TCRγδ^+^CD24^+^ thymocytes. (D) Representative flow cytometry plots and calculated frequencies of CD25^+^ and CD25^−^ CD24^+^ γδ thymocytes. (E) Analysis of CD73 and IFN‐γ expressing populations within CD25^−^CD24^−^ γδ thymocytes. (F) Representative flow cytometry of identifying γδ thymocytes and frequencies of total thymus and TCRγδ^+^ cellularities in C57BL/6 WT IFN‐γ^YFP^ (WT, black, *n* = 6) and *Ccl21a^−/−^* IFN‐γ^YFP^ (*plt/*plt, blue, *n* = 8) adult mouse thymus. (G) Total number of TCRγδ^+^CD24^+^ thymocytes. (H) Total frequencies of CD25^+^ and CD25^−^ CD24^+^ γδ thymocytes. (I) Analysis of CD73 and IFN‐γ expressing populations within CD25^−^CD24^−^ γδ thymocytes. In all cases, error bars represent mean ± SEM. For statistical analysis, a one‐way ANOVA with Tukey's multiple comparisons test was used in (A), an unpaired Student's *t*‐test was used in all other cases. To denote statistical significance; *p < 0.05; **p < 0.01; ***p < 0.001; ****p < 0.0001. Flow cytometry data representative of four independent experiments.

During αβT‐cell development in the adult thymus, expression of the chemokine receptor CCR7 is important for developing thymocytes gain access to medullary microenvironments [14]. To examine the signals that are provided by the thymus medulla to aid effector γδT‐cell development, we examined expression of CCR7 and investigated possible roles for its chemokine ligands CCL19 and CCL21. Flow cytometric analysis of adult CD24^+^ γδ thymocyte subsets showed that CCR7 was expressed by CD24^+^CD25^+^CD73^−^ progenitors (Fig. 3A). Moreover, more mature CD24^+^CD25^−^CD73^−^ and CD24^+^CD25^−^CD73^+^ subsets expressed higher levels of CCR7 expression (Fig. 3A), suggesting that CCR7 expression occurs before CD73 expression, and increases during intrathymic effector γδT‐cell development. Given that CD73 expression has been linked to γδTCR signaling, these data indicates that for γδT‐cells, CCR7 expression may occur prior to TCR signaling. Alternatively, CD73 may be a later marker of γδTCR signaling, which is preceded by CCR7 induction. Interestingly, a scenario where CCR7 expression occurs before CD73 expression in γδT‐cell development then contrasts with the timing and requirement for induction of CCR7 expression in the extrathymic maturation of mature αβT‐cells, where CCR7 expression in immature CD4^+^CD8^+^ thymocytes is induced following αβTCR‐mediated signaling for positive selection [15].

To examine the functional importance of CCR7‐CCR7 ligand interactions, we examined effector γδT‐cell development in *plt/plt* mice which lack expression of the CCR7 ligands CCL19 and CCL21, crossed to IFN‐γ^YFP^ GREAT reporter mice [16]. Here, we saw a reduction in the numbers of total γδTCR^+^ and CD24^+^γδTCR^+^ thymocytes in *plt/plt* adult thymus (Fig. 3B and C). Moreover, while we saw no significant change in the frequency of CD25^+^ precursors, we saw reduced numbers of CD25^−^ cells (Fig. 3D), which mapped to a reduction in both CD73^+^IFN‐γ^YFP−^ and CD73^+^IFN‐γ^YFP+^ effector subsets (Fig. 3E). These data indicates that absence of CCR7‐CCR7 ligand mediated migration is sufficient to significantly impair medulla‐dependent intrathymic effector lineage commitment of developing γδ thymocytes.

Given that *plt/plt* mice lack both CCL19 and CCL21, analysis of these mice does not allow for examination of whether there is a specific requirement for a particular chemokine during adult γδT‐cell development, or whether there is redundancy in CCR7 ligands. To address this, we examined intrathymic effector priming of adult γδT‐cells in *Ccl19^−/−^* mice and *Ccl21^−/−^* mice. In *Ccl19^−/−^* adult mice that lack CCL19 but retain CCL21, we saw no perturbations in CD24^+^ γδ thymocyte development, with numbers of CD25^+^ γδ progenitors, and CD25^−^CD73^−^ and CD25^−^CD73^+^ effector‐committed γδ T cells all comparable to WT controls (Supporting information Fig. S2A–D). In contrast, in *Ccl21a^−/−^* mice crossed with IFN‐γ^YFP^ GREAT reporter mice, where CCL21 is absent but CCL19 is present, we found intrathymic γδΤ‐cell development to be significantly impaired, with a phenotype closely resembling that seen in the *plt/plt* thymus. Specifically, we observed a reduction in total TCRγδ^+^ T‐cell numbers, which equated to a large reduction in the CD24^+^ newly produced fraction (Fig. 3F and G). Moreover, as in the *plt/plt* thymus, we saw no impact on CD25^+^ γδ progenitor development, but saw a significant loss of CD25^−^ γδ thymocytes, including both CD73^+^IFN‐γ^YFP−^ and CD73^+^IFN‐γ^YFP+^ effector subsets (Fig. 3H and I). Thus, these findings indicate that CCR7‐mediated migration plays an important role in effector γδT‐cell development in the adult thymus. Moreover, defects in γδT‐cell development in *Ccl21a^−/−^* mice, but not *Ccl19* ^−/−^ mice, demonstrate that CCL21, but not CCL19, is important in this process.

## Concluding remarks

The adult thymus supports the development of both naïve and effector primed γδT‐cells, but studies of this process are complicated by the heterogenous nature of the γδTCR^+^ population. Using CD24, Rag2GFP, CD73, and IFN‐γ^YFP^ GREAT, we identified γδ thymocytes undergoing intrathymic development to study the timing of intrathymic γδ effector priming and regulation of this process. We found that the development of CD73^+^ and CD73^+^IFN‐γ^YFP+^ γδ thymocytes required the presence of intact thymus medullary microenvironments, which correlated with a requirement for interactions between CCR7 and the medullary chemoattractant CCL21 but not CCL19. Interestingly, while previous studies suggested reduced γδT‐cell output from *Ccr7^−/−^* thymus [17], it remained unclear whether this was caused by an impact on γδT‐cell development, or mapped to specific roles for either CCL19 or CCL21. Indeed, our findings that CCR7‐CCL21 interactions are essential for the development of both CD24^+^CD73^+^ and CD24^+^CD73^+^IFN‐γ^YFP+^ γδ thymocytes perhaps suggest that these previous observations may be explained by a reduction in intrathymic development.

TCR‐ligand engagement is required for the development of IFN‐γ‐producing γδ thymocytes, and in its absence in the adult thymus, γδTCR cells remain naïve [3, 5]. This suggests that the requirement for the thymus medulla during adult intrathymic γδT‐cell priming may involve the provision of γδTCR ligands by cells that reside within, or form, thymic microenvironments, including mTEC and/or DCs. Interestingly, previous studies on embryonic γδ thymocyte development showed that mTEC‐γδ thymocyte crosstalk controls effector priming of IFN‐γ‐producing DETC [18]. Currently, it is not clear how mTEC‐γδ thymocyte interactions might influence effector priming in the adult thymus. Interestingly, during the development of CD1d‐restricted iNKT‐cells, an αβT‐cell subset that acquires intrathymic effector function, mTEC are known to be involved via their production of cytokines that include IL25 and IL15 [19]. Moreover, lymphotoxinβ receptor signaling plays an important role in iNKT‐cell development and represents a pathway previously linked to γδT‐cell development [19, 20]. Finally, it is perhaps of note that thymocyte–mTEC interactions are frequently reciprocal, with thymocyte development driving mTEC maturation via a crosstalk mechanism involving multiple TNFR superfamily members that include RANK‐RANKL interactions [21]. While RANKL is expressed during embryonic γδT‐cell development [18], whether RANK‐RANKL is involved during medullary maturation of adult γδT cells is not clear. Further work is required to examine whether the requirement for the adult thymus medulla during effector priming of γδT‐cells has parallels with events that control iNKT‐cell development in the adult thymus and/or intrathymic effector priming of embryonic γδT cells.

In conclusion, our study provides further understanding of the regulation of intrathymic effector‐priming of IFN‐γ‐producing γδ thymocytes during their development in the adult thymus and demonstrates that access to signals within the medulla is an important process of the intrathymic effector priming mechanism. Furthermore, by identifying a specific role for CCL21 but not CCL19, we reveal non‐redundancy in the requirement for CCR7 ligands in this process.

## Materials and methods

### Mice

The following adult mice were used, aged between 8 and 12 weeks old, mixed sexes: wild‐type (WT) C57BL/6, *plt/plt* [22], *Ccl19* ^−/−^ [23], *Ccl21a* ^tdTom^ knock‐in [24], and *Relb^−/−^* [25]. CD45.1^+^ BoyJ mice were used as hosts for BM chimeras. C57BL/6, *plt/plt*, and *Ccl21a^tdTom^* mice were crossed with IFNγ^YFP^ GREAT [11] reporter mice to detect IFNγ production and were also used as hosts for grafting experiments. C57BL/6 mice were also crossed with Rag2GFP reporter mice [8]. All strains were housed within the Biomedical Services Unit at the University of Birmingham.

### Flow cytometry

Single cell thymocyte suspensions generated by mechanical dissociation of the thymus lobes were stained with antibodies to the following (sourced from eBioscience and BioLegend unless otherwise indicated): CD3ε (BV711; Clone 17A2 and PE; Clone 145‐2C11), TCRβ (APC eFluor 780; H57.597), TCRγδ (BV421; Clone GL3), CD24 (PerCP Cy5.5; Clone M1/69), CD25 (BV650; Clone PC61), CD73 (BV605; Clone TY/11.8), CCR7 (PE; Clone 4B12), Rat IgG1 isotype (BV605; Clone RTK2071), and Rat IgG2aκ isotype (PE; Clone eBR2a). Viable cells were distinguished from dead cells using the Fixable live/dead ZombieAqua 510 (BioLegend). Samples were enriched for γδ T‐cells to improve analysis of rarer populations by depleting TCRβ^+^ cells using anti‐TCRβ PE and then incubating the samples with anti‐PE microbeads and deplete using LD columns (Miltenyi Biotec).

### Thymus transplantation

Thymus lobes were harvested from C57/BL6 and *Relb^−/−^* embryonic day 15 mice and cultured in fetal thymic organ culture conditions with 2‐dGuo to deplete thymus lobes of hematopoietic cells. These lobes were transplanted under the kidney capsule of WT IFN‐γ^YFP^ GREAT reporter mice, as previously described [13], and recovered after 6 weeks to analyze γδ T‐cell development within the grafts.

### Statistical analysis

Prism 9 (GraphPad Software) was used to perform all statistical analyses. To compare multiple populations, a one‐way ANOVA test was used, in all other cases an unpaired Student's *t* test was used. Graphs were annotated with the following indicators to signify statistical significance: *p < 0.05; **p < 0.01; ***p < 0.001; and ****p < 0.0001. Nonsignificant differences were not specified. In all figures, bar charts and error bars represent means ± SEM, respectively.

## Conflict of interest

The authors declare no commercial or financial conflict of interest.

## Ethics approval

Husbandry, housing, and experimental methods involving mice were performed at the Biomedical Services Unit at the University of Birmingham in accordance with the local Ethical Review Panel and U.K. Home Office Regulations (Animal project License no. P3ACFED06, PP7518148, and PP2990911).

### Peer review

The peer review history for this article is available at https://publons.com/publon/10.1002/eji.202350388

**Abbreviation**
mTEC: medullary thymic epithelial cells

## Supporting information

Supporting Information

## Data Availability

The data that support the findings of this study are available from the corresponding author upon reasonable request.
