# Supplementary material for: The medulla controls effector primed γδT‐cell development in the adult mouse thymus
Source: Eur J Immunol. 2023 Mar 28;53(6):2350388. doi: 10.1002/eji.202350388 (PMC10947249; doi:10.1002/eji.202350388)
Supplement: Supplementary file 1 — Supporting Information [file EJI-53-0-s001.pdf]

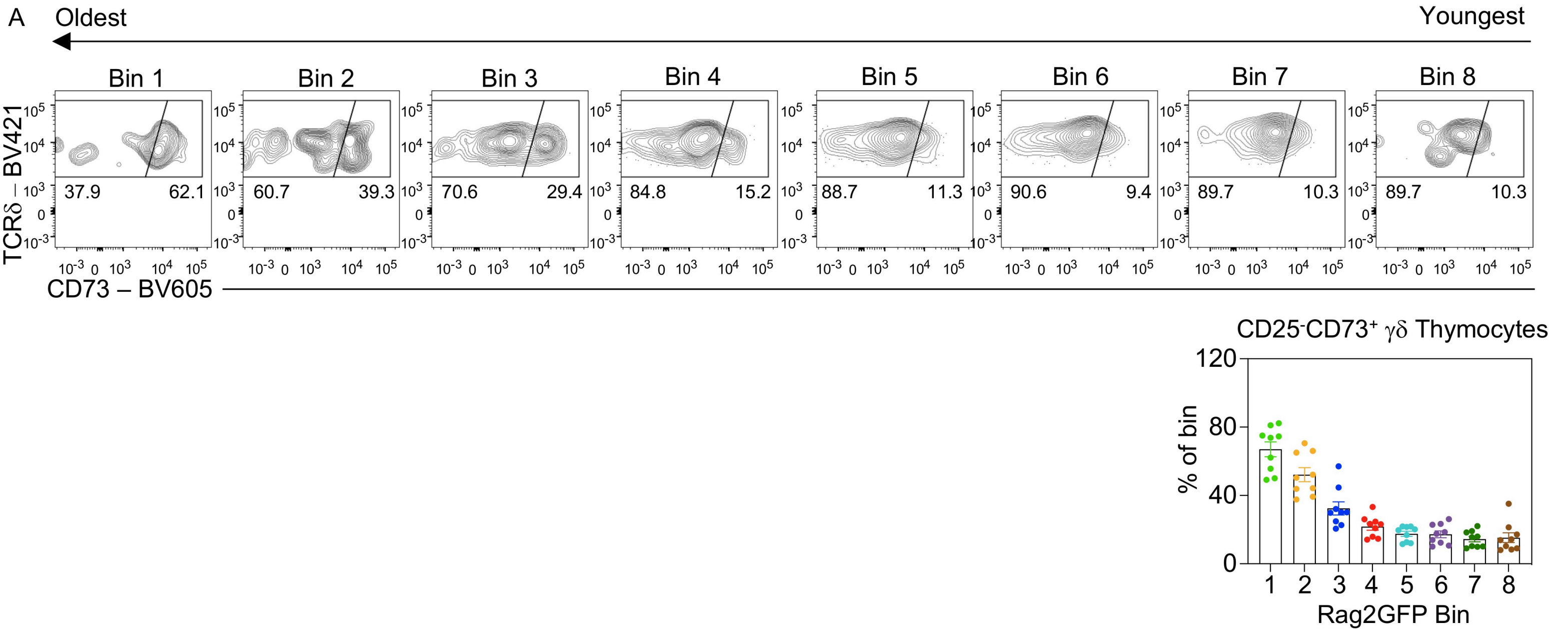
A
Oldest
Youngest
Bin 1
Bin 2
Bin 3
Bin 4
Bin 5
Bin 6
Bin 7
Bin 8
37.9
62.1
60.7
39.3
70.6
29.4
84.8
15.2
88.7
11.3
90.6
9.4
89.7
10.3
89.7
10.3
TCRδ – BV421
CD73 – BV605
CD25-CD73+ γδ Thymocytes
% of bin
Rag2GFP Bin

Supplementary Information Figure 1. Developmental Progression of Rag2GFP^{+}CD24^{+}CD25^{-} CD73^{-} and CD73^{+} $\gamma\delta$ Thymocyte Subsets

(A) Bins of Rag2GFP expression within Rag2GFP^{+}CD24^{+}CD25^{-} $\gamma\delta$ thymocytes are displayed from lowest Rag2GFP expression (Bin 1) to highest Rag2GFP expression (Bin 8) and plotted for TCRδ and CD73. The frequency of CD25^{+}CD73^{+} from each of the 8 bins is displayed. In all cases, error bars represent mean +/- SEM. Flow cytometry data representative of 4 independent experiments, total n=9.

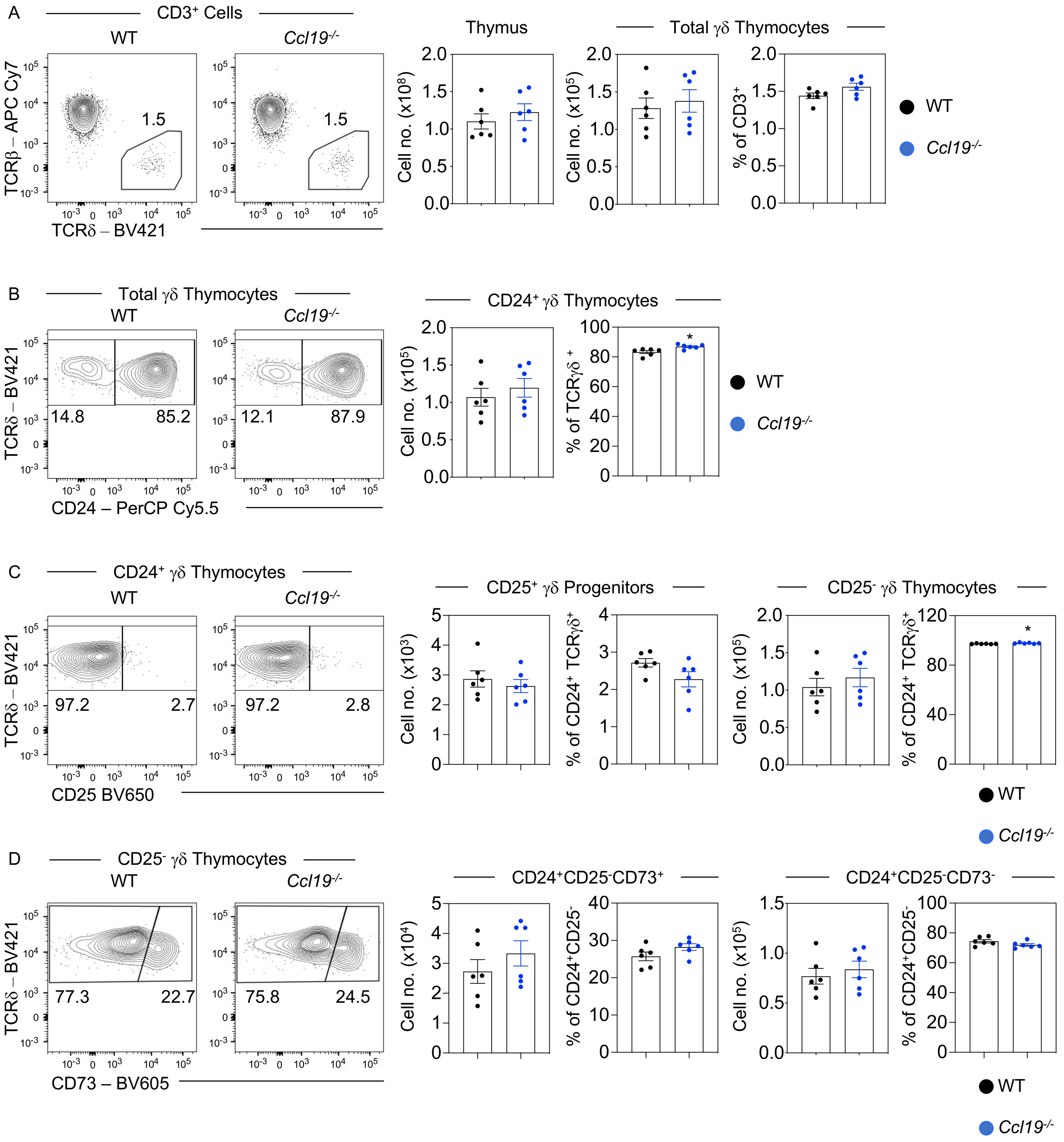
A
CD3+ Cells
WT
Ccl19-/-
TCRβ – APC Cy7
TCRδ – BV421
1.5
1.5
Thymus
Total γδ Thymocytes
Cell no. (x10^8)
Cell no. (x10^5)
% of CD3+
WT
Ccl19-/-
B
Total γδ Thymocytes
TCRδ – BV421
CD24 – PerCP Cy5.5
14.8
85.2
12.1
87.9
CD24+ γδ Thymocytes
Cell no. (x10^5)
% of TCRγδ+
*
C
CD24+ γδ Thymocytes
TCRδ – BV421
CD25 BV650
97.2
2.7
97.2
2.8
CD25+ γδ Progenitors
CD25- γδ Thymocytes
Cell no. (x10^3)
% of CD24+ TCRγδ+
Cell no. (x10^5)
% of CD24+ TCRγδ+
*
D
CD25- γδ Thymocytes
TCRδ – BV421
CD73 – BV605
77.3
22.7
75.8
24.5
CD24+CD25-CD73+
CD24+CD25-CD73-
Cell no. (x10^4)
% of CD24+CD25-
Cell no. (x10^5)
% of CD24+CD25-

Supplementary Information Figure 2. γδ Thymocyte Development Is Unperturbed In The Absence Of CCL19

Analysis of TCRγδ$^+$ thymocyte development in C57BL/6 WT (WT, black, n=6) and *Ccl19*$^{-/-}$ (blue, n=6) adult mouse thymus. (A) Representative flow cytometry plots and frequencies of total thymic cellularity and TCRγδ$^+$ thymocytes. (B) Representative flow cytometry plots and frequencies of TCRγδ$^+$CD24$^+$ newly produced γδ thymocytes and (C) break down into subsequent populations of CD25$^+$ γδ progenitors and CD25$^-$ cells. (D) Representative plots of CD73 expression within the TCRγδ$^+$CD24$^+$CD25$^-$ population to identify CD73$^-$ uncommitted/naïve and CD73$^+$ effector primed γδ thymocytes. Error bars represent mean +/- SEM. Flow cytometry data is representative of 2 independent experiments.
